# Polymer-Drug Conjugate, a Potential Therapeutic to Combat Breast and Lung Cancer

**DOI:** 10.3390/pharmaceutics12050406

**Published:** 2020-04-29

**Authors:** Sibusiso Alven, Xhamla Nqoro, Buhle Buyana, Blessing A. Aderibigbe

**Affiliations:** Department of Chemistry, University of Fort Hare, Alice Eastern Cape 5700, South Africa

**Keywords:** breast cancer, lung cancer, chemotherapy, polymer-based carriers, polymer-drug conjugates

## Abstract

Cancer is a chronic disease that is responsible for the high death rate, globally. The administration of anticancer drugs is one crucial approach that is employed for the treatment of cancer, although its therapeutic status is not presently satisfactory. The anticancer drugs are limited pharmacologically, resulting from the serious side effects, which could be life-threatening. Polymer drug conjugates, nano-based drug delivery systems can be utilized to protect normal body tissues from the adverse side effects of anticancer drugs and also to overcome drug resistance. They transport therapeutic agents to the target cell/tissue. This review article is based on the therapeutic outcomes of polymer-drug conjugates against breast and lung cancer.

## 1. Introduction

Cancer is a chronic disease that leads to great mortality around the world and cancer cases are rising continuously [1]. It is the second cause of death worldwide, followed by cardiovascular diseases [2]. It is characterized by an abnormal uncontrolled proliferation of any type of cells in the human body [3]. It is caused by external factors, such as smoking, infectious organisms, pollution, and radiation; it is also caused by internal factors, such as immune conditions, hormones, and genetic mutation [3]. Although there are several types of cancer, the most common ones are breast, colorectal, and lung cancer [4]. The most-reported common cancer in women is breast cancer, while in men, it is lung cancer. It has been estimated that the cases of cancer burden in the world increased to 18.1 million, while deaths caused by cancer increased to 9.6 million in 2018 [5].

Treatment of cancer includes radiotherapy, surgery, hormonal therapy, immunotherapy, and chemotherapy (anticancer drugs) [3,6]. The method of treatment employed depends on the location and the stage of the tumor [7]. The use of anticancer drugs is the most employed method, and they are therapeutic agents that can be used to target proteins, tissue environment, and genes that are responsible for cancer growth. Cancer is treated by combination therapy, involving the use of two or more anticancer agents [8]. In addition to combination therapy, a polymer-based drug delivery system is another potential strategy that has been reported to enhance the therapeutic efficacy of anticancer agents [9].

Polymer-based drug delivery systems have been utilized in biomedical applications to deliver therapeutic agents to the target biological environment [10]. They exhibit distinct features, such as reduced drug toxicity, improved patient compliance, increased drug solubility, enhanced drug bioavailability, biocompatibility, and biodegradability, control drug release mechanism, protect the drug from deactivation, and preserve drug activity during circulation [11]. There are several polymer-based drug delivery systems that have been formulated to improve therapeutic outcomes of anticancer drugs, such as polymer capsules [12,13,14,15,16,17], polymeric nanoparticle [18,19,20,21,22,23], dendrimers [24,25,26,27,28,29], micelles [30], hydrogels [31], nanogels [32], in situ gels [33], polymer-drug conjugates [34,35,36,37], and nanoliposomes [38,39,40,41,42,43]. This review article is focused on polymer-drug conjugates, which were recently reported between 2016 and 2019 with good therapeutic efficacy against breast and lung cancer, which are the most common forms of cancer in women and men, respectively.

## 2. Classification of Anticancer Chemotherapeutics Based on Their Mechanism of Actions

**Figure 1:**
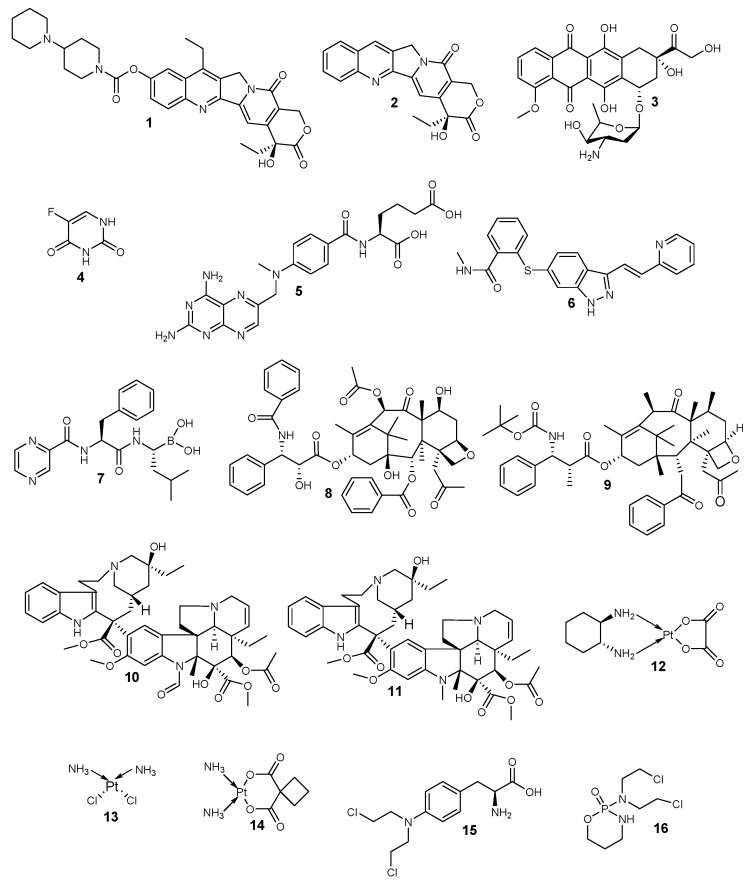
Anticancer drugs based on their mode of action.

Anticancer drugs are categorized based on their mechanism of action into four distinct classes: topoisomerase inhibitors, antimetabolites, anti-tubulin agents, and alkylating agents (Figure 1 and Table 1). Topoisomerase inhibitors hinder the replication of deoxyribonucleic acid (DNA), and their examples include irinotecan **1**, camptothecin **2**, and doxorubicin **3** [44,45,46]. They act by binding to the topoisomerase active site resulting in the hindrance of the binding of the DNA substrate. They also form a cleavage complex, which prevents enzyme turnover and the build-up of high levels of the cytotoxic cleavage complex within the cell [47].

Antimetabolites hinder biosynthesis of nucleic acids, and the examples of antimetabolites are 5-fluorouracil **4**, methotrexate **5**, bevacizumab **6**, and bortezomib **7** [48]. Anti-tubulin agents disrupt mitotic spindles and terminate mitosis [49]. Examples of anti-tubulin agents include paclitaxel **8**, docetaxel **9**, vincristine **10**, and vinblastine **11**, and they act on tubulins [50]. Paclitaxel is the most active anticancer drug that is employed for the treatment of several types of cancer. Alkylating agents covalently bind with the DNA and crosslink them [51]. Examples of alkylating agents are oxaliplatin **12**, cisplatin **13**, carboplatin **14**, melphalan **15**, and cyclophosphamide **16**, which result in DNA disruption [51].

### Limitations of Anticancer Drugs and Multi-Drug Resistance

The aforementioned anticancer drugs indicated in Table 1 are used to target the quick and rapid division of cells. There are several cells in the body that divide rapidly in a normal way, such as the hair follicle cells, bone marrow cells, and digestive tract cells, which are also affected unintentionally by those anticancer chemotherapeutics and result in severe side effects [52]. The side effects that can be caused by anticancer drugs include neurotoxicity, cardiotoxicity, mucositis, myelosuppression, and immunosuppression [53]. Furthermore, the widespread distribution and very short half-life of drugs require more dosing of the anticancer drugs, which can lead to an increase in the aforementioned side effects. Another limitation of most of the presently used anticancer drugs is their hydrophobic nature making them insoluble in aqueous media. The major limitation of all anticancer drugs is drug resistance caused by various factors, including several mutations, etc. [54].

The multidrug resistance (MDR) is the main problem that makes the treatment of cancer challenging. MDR has been reported due to mechanisms, such as the cancer cells that survived the chemotherapeutics, the pumping out of chemotherapeutics by transporters known as ATP-binding cassette (ABC), cell defense mechanism, the evolving adaptation of the cancer cells biological environment, the mutation of oncogenes that are resistant to former treatments that were employed [55]. The overexpression of the transporters, such as ABCB1, known as P-glycoprotein, P-gp, ABCG2, known as breast cancer resistant protein, BCRP, and ABCC1, known as multidrug resistance-associated protein 1, MRP1, contributes to MDR [55]. They expel drugs away from the cell, resulting in decreased intracellular accumulation. The strategy that has been proposed to combat P-glycoprotein 1 is the co-administration of anticancer drugs with P-glycoprotein 1 inhibitor loaded in nanoparticles [56]. Although this strategy is a potential approach to overcome drug resistance, it has a limitation, which is the lower capability of some anticancer drugs to cleave from the nanoparticles or not cleaving at all [7].

The biological features of tumors that hinder the accessibility of drugs and protect the cancer cells from drug cytotoxicity are tumor vasculature, which is abnormal and heterogeneous, cell density, interstitium, and interstitial fluid pressure [57]. The immature vasculature of tumors hinders the delivery of nutrients and oxygen to the cancer cells. The poor supply of nutrients, oxygen, etc. to the tumor cells induces a mechanism known as angiogenesis in which new lymphatic and blood vessels are formed. The acidic microenvironment of the tumor promotes tumor cell dissemination. The adaptation of the cancer cells contributes to the maintained acidic pH of the tumor [58]. Selected anticancer drugs are easily ionized, resulting in their poor diffusion via the cell membranes. Low pH has been reported to result in tumor resistance to weak basic anticancer drugs [58]. The application of polymer-based drug delivery systems is one potential approach that can combat the aforementioned limitations of anticancer chemotherapy, including multidrug resistance.

## 3. Polymer-Drug Conjugates

**Figure 2:**
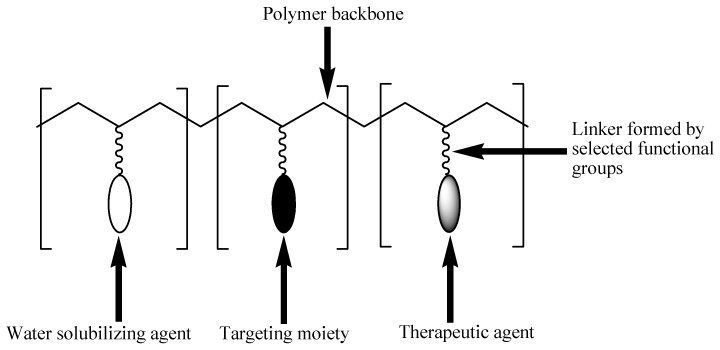
Schematic diagram of the Ringsdorf model of polymer-drug conjugates.

Polymer-drug conjugates, which are also known as polymeric prodrugs, are drug delivery systems that are formulated for the incorporation of therapeutic agents into polymers of choice using selected functionalities [59]. These delivery systems are unique and composed of three units: solubilizing unit, targeting moiety, and a therapeutic agent. These units are covalently incorporated into the polymer backbone. This model was first proposed by Helmut Ringsdorf in 1975 (Figure 2) [60].

The solubilizing unit enhances the water solubility of the conjugates. The targeting moiety improves the transportation of the conjugate to the targeted cell/tissue [61]. Some targeting moieties used include folic acid, engineered antibodies, sugars, and peptides. Their capability to interact exclusively with specific receptors present on selected cell types makes them very important in the development of polymer-drug conjugates [62]. The therapeutic agent is usually incorporated into the polymeric backbone via a linker [61]. The linker plays an important role in the release of the conjugated drug under certain conditions, such as a change in pH, the presence of enzymes or sensitivity, to overexpressed groups of diseased organ/tissue [63]. The type of linkers used influence the % loading of the drug to the carrier, drug stability, and drug release mechanism. Several linkers, such as hydrazine, azo, peptides, disulfide, etc. have been used for drug conjugation to polymers [64].

There are three known synthetic routes for the preparation of polymer-drug conjugates, such as the incorporation of a drug to a synthesized polymeric carrier, the incorporation of a drug to a monomer before polymerization, and the incorporation of the drug as monomers or initiators during the polymerization reaction [65].

The preparation of polymer-drug conjugates via the incorporation of a drug to a monomer before polymerization has been reported to overcome the problem of uncontrolled conjugation to the polymer backbone resulting in a high drug loading and controlled drug loading. The conjugation of the drug to the monomer does not interfere with the polymerization reaction, and the problem of steric hindrance during the conjugation is also overcome [65,66].

Polymerization reactions, such as ring-opening metathesis polymerization (ROMP), ring-opening polymerization (ROP), reversible addition-fragmentation transfer polymerization (RAFT), have been used for the preparation of polymer-drug conjugates, whereby the drug is conjugated first to the monomer [65,67,68,69].

The conjugates prepared via the aforementioned method displayed a good feature, which includes triggered drug release suitable for conjugates loaded with multiple drugs. However, some polymer-drug conjugates prepared by ROMP and RAFT displayed a non-biodegradable backbone [65,69,70]. Employing ROP has been reported to result in polymer-drug conjugates with a biodegradable backbone [70]. It is important to mention that using a drug as a monomer is not a suitable approach for many drugs, even though it increases the drug conjugation in the polymeric carrier significantly.

Some of the functional groups suitable for the conjugation of drugs in polymer-drug conjugates include amines, alcohols, and carboxylic groups etc. [71]. Polymer-drug conjugates can be prepared using branched or linear polymeric carriers. Linear carriers include polyaspartimide, poly(malic acid), poly(vinyl pyrrolidone), poly(ethylene glycol) (PEG), and poly(vinyl alcohol) (PVP) polymers [72]. Branched carriers include poly(amidoamine) and poly(ethyleneimine) polymers [71]. There are several advantages that are displayed by polymer-drug conjugates, such as improved drug bioavailability and biodegradability [73], reduced drug toxicity [74], improved drug stability and water solubility, enhanced drug biocompatibility and drug delivery by sustaining and controlling the drug release mechanism [75], and they have the ability to overcome drug resistance. However, polymer-drug conjugates have few limitations in combination therapy, such as difficulties in the identification of ratios of incorporated therapeutic agents and poor drug loading capacity [76]. The conventional anticancer drugs suffer from pharmacological limitations, such as poor oral bioavailability; poor water solubility in which most of them are hydrophobic, and as such, they can not penetrate the biological membranes; instability in circulation and short circulation time due to some of them being engulfed by the macrophages, and thus their short circulation time makes their interaction with the cancerous cells ineffective; poor selectivity towards cancer cells resulting in the normal cells being exposed to the toxic side effects of the drugs; overexpression of a multidrug resistance protein, P-glycoprotein on the surface of the cancerous cells, which act as an efflux pump, preventing the accumulation of anticancer drugs inside the tumor resulting in drug resistance [77,78,79].

### Physicochemical Properties of Polymer-Drug Conjugates for Enhanced Tumor Uptake

The physicochemical properties of polymer-drug conjugates, such as the surface charge, conformation, size, and biocompatibility, influence factors, such as their absorption, distribution, excretion, etc. [65].

Cancer cell surfaces have been reported to be negatively charged resulting from the movement of negatively charged constituents, such as phosphatidylserine, anionic phospholipids, glycoproteins, etc. to the cell surface of cancer [80]. The pH of most solid tumors is in the range of 5.7–7.8 [81]. Tumor tissues display acidic plasma pH when compared to normal tissues, which exhibit alkaline-outside pH gradients [82], resulting in the significantly lower extracellular pH of malignant tumors when compared to normal tissues [83]. Positively charged nanoparticles are preferentially taken up by the tumors [84,85], and they are retained over an extended period when compared to negatively charged or neutral nanoparticles [84,86]. Positively charged nanoparticles can penetrate the tumor deeper when compared to anionic and neutral nanoparticles [65,87,88,89]. Cationic drug carriers improve the interaction of anticancer drugs with transmembrane receptors, such as integrins, which are involved in cell invasion and metastasis and in signaling processes [90].

The uptake of nanoparticles by the tumor tissue is also influenced by the particle size. Nanoparticles, which are small-sized, penetrate deeper into the tumor tissue and are not cleared rapidly from the tumor [65]. However, the design of small-sized nanoparticles is challenging, and they do not exhibit high drug loading capability. In order to overcome the above-mentioned challenge, stimuli-responsive nanoparticles that have the capability to shrink their sizes for enhanced tumor penetration have been developed.

Nanocarriers with sizes below 100 nm are suitable for systemic distribution. In order to avoid the rapid clearance of the nanocarriers by the kidneys, the particle size of nanocarriers should be larger than 6 nm. However, some polymer-drug conjugates in clinical trials have been reported to be below 10 nm [91]. During circulation, nanocarriers can bind with the proteins in the blood, leading to aggregation, and they can be taken up by the macrophages. The aforementioned factor reduces the amount of the drug that is taken up into the tumor tissue. The particle size of the conjugates determines their penetration into tumor tissue. Selected particle sizes have been reported to enhance tumor tissue penetration when compared to larger particle sizes [92,93]. Sub-100 nm is a size range of nanocarriers suitable for passive tumor targeting, which is also influenced by their composition [94].

The molecular weight of polymer conjugates has been reported to influence their circulation in vivo. Polymer-drug conjugates with high molecular weight display extended intravascular half-life with slower excretion from the tumor and body [65,95]. Researchers have reported the high tumor accumulation of high-molecular-weight greater than 50 kDa in clinical trials [96,97,98]. However, high-molecular-weight polymers can also hinder conjugated drugs from reaching their target cell/tissue [97]. Lower molecular weight polymer-drug conjugates have also been reported for combination therapy and enhanced cellular uptake [99,100].

**Figure 3:**
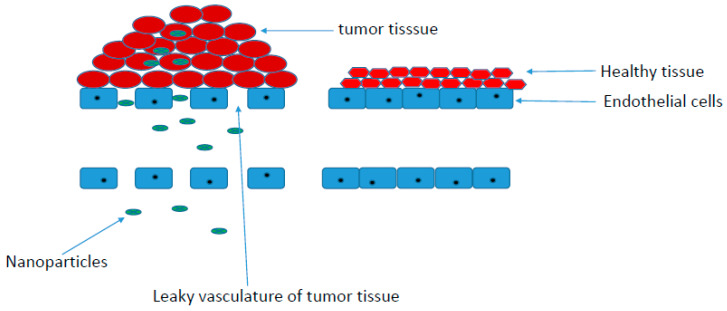
EPR (enhanced permeation retention) uptake of polymer-drug conjugates.

Tumors consist of vasculature and supporting cells. Their capillaries are irregular when compared to a normal tissue vasculature, which is characterized by tight endothelial cells, preventing the penetration of nanoparticles [101]. Tumor tissue vasculatures are leaky and highly permeable. Their permeability promotes enhanced permeation retention (EPR) accumulation effects of polymer-drug conjugates in solid tumors. The accumulation of polymer-drug conjugates in the tumor is influenced by the high interstitial fluid pressure in tumor tissues when compared to the normal tissues (Figure 3) [101]. EPR-mediated accumulation of nanoparticles has been reported to be high in tumors, such as breast, lung, and ovary tumors when compared to other tumors [102].

**Figure 4:**
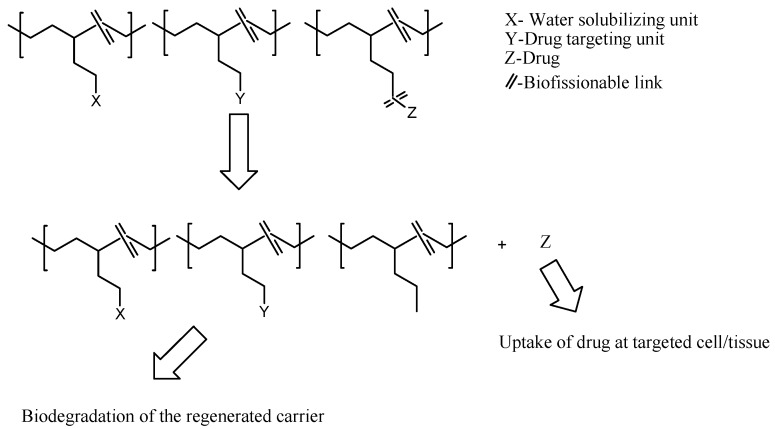
In vivo pharmacokinetic release of drug from the polymer-drug conjugates.

Active targeting of nanoparticles can also be influenced by the conjugation of targeting molecules overexpressed on the tumor cell surface [103]. The targeting molecules conjugated on nanoparticles bind to cells via an endosome-dependent mechanism. This mechanism bypasses the drug efflux pump resulting in a high intracellular uptake [101]. Drug release from polymer-drug conjugates resulting from the biological stimulus, such as enzyme or pH, has been widely reported by employing stimulus sensitive linkers, which trigger drug release (Figure 4) [104,105]. Some examples of pH-sensitive linkers are hydrazine, amide, imine, cis-aconityl, oxime, thiopropionate, etc. [65].

## 4. Polymer-Anticancer Drug Conjugates Effective against Breast Cancer (In Vivo and In Vitro)

Breast cancer progresses from the breast tissue. This type of cancer is the most commonly diagnosed cancer, and it results in 25% of all cases, and it causes 15% of cancer deaths in women worldwide [106]. The triple-negative breast cancer (TNBC) is the most severe subtype of breast cancer, and it lacks the expression of human epidermal growth factor receptor 2 (HER2), estrogen receptor (ER), and progesterone receptor (PR) [106]. Symptoms of breast cancer may include a lump in the breast, dimpling of the skin, change in the shape of the breast, red patch of the skin, swollen lymph nodes, shortness of breath, bone pain, discharge of fluid from the nipple, and change in breast size [107]. Breast cancer is caused by factors, such as lack of exercise, obesity, smoking, ionizing radiation, menstruation at an early age, hormone replacement therapy during menopause, family genetic transmission, etc. [108]. The currently used breast cancer therapy includes monoclonal antibody therapy, immunotherapy, hormone therapy, radiation therapy, surgery, and chemotherapy [109]. There are several chemotherapeutic agents that are useful for the treatment of breast cancer, including paclitaxel, docetaxel, doxorubicin, and platinum agents (e.g., cisplatin, etc.) [110]. They all suffer from severe pharmacological limitations, and hence, there is an urgent need to enhance their chemotherapeutic outcomes by incorporating them into the polymers to form polymer-drug conjugates. Researchers have developed polymer-drug conjugates effective against breast cancer in vitro and in vivo (Table 2).

Cai et al. prepared polymer-drug conjugates incorporated with paclitaxel as an anticancer agent [111]. The drug was incorporated into the polymer backbone via an enzyme-sensitive tetrapeptide linker. The biodegradability studies and drug release profiles showed that these polymer-drug conjugates linker promoted the degradation of the conjugate with a high molecular weight to low molecular weight products followed by the release of the drug in the cancer microenvironment. After the release of paclitaxel, its stability was maintained in the circulation system of the blood. The in vivo studies of the conjugates in mice bearing 4T1 breast cancer model revealed a 16.7% tumor growth inhibition (TGI), leading to tumor weight of 841 mg when the free drug was administered. The conjugates loaded with nanoparticles and paclitaxel displayed a significant 95.5% TGI, leading to a tumor weight of 45.3 mg. The conjugates significantly inhibited proliferation and induced apoptosis of the 4T1 murine breast cancer cells in the xenograft tumor model with no side effects [111].

Vogus et al. formulated hyaluronic acid polymer-drug conjugates incorporated with doxorubicin (DOX) and gemcitabine (GEM) for synergistic effect against triple-negative breast cancer. The drugs were incorporated into the same polymer by conjugating the adipic acid dihydrazide linker followed by GEM prodrug, and DOX via the hydrazone bond, respectively. The in vitro drug release mechanism of both chemotherapeutics from the conjugates showed a fast release of GEM with more anticancer activity against breast cancer cells when compared to the slow-release mechanism of doxorubicin polymeric prodrugs in vitro. Furthermore, the conjugates loaded with both drugs were active in inhibiting the formation of an orthotopic, aggressive 4T1 tumor model in vivo when compared to the free doxorubicin and polymer-conjugates loaded with a single drug [112]. 4TI tumor model was used to study the spreading of cancer metastases to local lymph nodes and distant organs due to the disease progression in human breast cancers. The conjugate containing both drugs was slightly toxic when compared to the polymer conjugate of either DOX or GEM, resulting in a low combination index of 0.42. Hyaluronic acid degraded easily in the lymphatic system, indicating that the subcutaneous administration of the conjugates resulted in the reduction of the molecular weight before its uptake into the systemic circulation. The administration of the conjugates subcutaneously in the mammary fat pads promoted the draining of the conjugates into the lymph nodes, which are usually the site of early tumor metastases. Controlling the release rate of multiple drugs from the same polymer backbone impact positively on the therapeutic efficacy of polymer-drug conjugates [112].

Kumar and co-workers prepared and performed in vivo and in vitro studies on trastuzumab, and folic acid conjugated multiblock copolymeric carriers for breast cancer cells targeting, employing ring-opening polymerization of lactide with polyethylene glycol to produce a triblock copolymer. Isomerization reaction was performed on the triblock copolymer. Folic acid was conjugated via the hydroxyl group on the multiblock polymer. The nanosize range of the polymer-drug conjugate nanoparticles was approximately 110 nm, with a good drug loading capacity of 22%. The in vitro drug release mechanism studies revealed their pH sensitivity with the drug release of 72% at pH 5.5 when compared to drug release of 18% at pH 7.4. The in vitro cellular uptake was 22% for polymeric prodrugs conjugated with both drugs (trastuzumab and folic acid) when compared to non-targeted polymeric prodrugs. Fluorescence-activated cell sorting (FACS) assessments on conjugated polymeric prodrugs showed interesting apoptosis of 80% when compared to non-conjugated polymeric prodrugs that showed a 20% in MCF-7 breast cancer cell lines [113].

Arminan et al. prepared and performed in vitro and in vivo studies on polymer-drug conjugates loaded with DOX utilizing N-(2-hydroxypropyl) methacrylamide (HPMA) as polymer, for breast cancer cells targeting. The overall studies of DOX-HPMA conjugates demonstrated reduced glycolysis, increased apoptosis, and reduced degrees of phospholipids when compared to the free DOX. The in vitro cytotoxicity evaluations of DOX-HPMA utilizing MTT assay displayed a significant cytotoxic efficacy against MCF7 breast cancer cells with a 50% inhibitory concentration (IC_50_) value of 7.5 µg/mL. However, the free DOX IC_50_ value was 5.2 µg/mL, suggesting that the free drug was more cytotoxic when compared to the conjugate. Clonogenic assay, together with the cell counts, revealed the cytotoxic outcome of both molecules and survival response absence in MCF7 human breast cells due to the induction of cell death, revealing the anticancer activity of DOX-HPMA. The in vivo studies on the 4T1 breast tumor mouse model using the conjugate revealed a high reduction in the growth of tumors when compared to free DOX-treated mice [114].

Arroyo-Crespo and co-workers prepared and characterized a pH-sensitive poly-L-glutamic acid (PGA)-drug conjugates to improve the anticancer efficacy of DOX and aminoglutethimide (AGM) utilizing two hydrazone linkers [115]. The low DOX loading incorporated via short length hydrazone spacers provided an improved anticancer activity on primary tumor growth and the toxicological profile in a murine model of triple-negative breast cancer [115]. A pH-sensitive linker, such as a hydrazone moiety or complex EMCH [*N*-ε-maleimidocaproic acid hydrazide] moiety, was used. The conjugates loaded with low DOX displayed a 50% reduction of the tumor in vivo when compared to PBS-treated mice, while the high loading DOX combination conjugates displayed a low antitumor activity when compared to the PBS-treated mice. The single conjugate loaded with DOX or AGM displayed no important anticancer activity in vitro [115]. The conjugates target the tumor microenvironment, thereby inducing mechanisms that promote tumor cell death and inhibit metastasis and cell proliferation. However, the conjugates with a hydrazine linker enhanced a significant immune response that resulted in a high survival rate in vivo [115].

Gu et al. investigated the efficacy of the delivery of anticancer drug to the affected milk duct, thereby reducing toxicities by employing polymer-drug conjugates. The common approach to the treatment of breast cancer is surgery and radiation. Poly(ethylene) glycol-doxorubicin conjugate nanocarriers of varying molecular sizes and architectures were prepared. In vitro cytotoxicity studies of the conjugates against MCF7 human breast cells showed IC_50_ values of 1.76, 3.86, 8.96, 18.11, 1.23, and 3.49 μM for the linear conjugates with molecular sizes of 5, 10, 20, 40 kDa, 4-arm 40 kDa, and 8-arm 40 kDa, respectively. The IC_50_ values revealed that the conjugates were less active when compared to the free drug that exhibited an IC_50_ value of 0.14 μM. The in vivo mammary gland retention studies in female Sprague-Dawley via intraductal administration into the mammary glands of the rat displayed longer half-life of the 40 kDa polymer-DOX conjugate when compared to the free DOX, 5 and 20 kDa polymer-drug conjugates. The mammary gland retention studies indicated that polymer-drug conjugates with larger hydrodynamic radii were retained longer in the mammary gland [116]. The increased molecular weight and decreased branching of the conjugates influenced the drug retention in the mammary gland. The administration of free drug intraductally resulted in severe side effects, such as ductal damage and severe inflammation. The PEG-DOX conjugates administration in vivo did not result in severe side effects. However, damaged epithelial cells were reported. The 40 kDa 4-arm PEG-DOX conjugates ductal retention half-life was the longest, and it was the most potent conjugate. The findings suggest that the administration of polymer-drug formulations intraductally can prolong ductal retention, resulting in reduced toxicity and enhanced sustained therapeutic outcomes [116].

Zhou et al. synthesized polymer-drug conjugates incorporated with podophyllotoxin (PPT), a chemotherapeutic agent, via ester bonds using poly(l-glutamic acid)-g-methoxy poly(ethylene glycol) [117]. The in vitro cytotoxicity studies on the conjugates displayed resistance index (RI) of 374 and 122.3 against MCF-7 breast cells in 72 h, respectively. The RI value of free PPT was 1 and 10.3 against MCF-7 cells and Paclitaxel-resistant (PTX) (A549) cells, respectively. The RI value for PPT-incorporated polymer prodrugs was 2.12 and 1.10 against MCF-7 cells and PTX (A549) cancer cells, respectively, demonstrating that PPT and PPT-incorporated polymer prodrug are potent in inhibiting in vitro P-gp overexpressed MDR cancer cell lines [117]. The single PPT and PPT-incorporated polymer prodrug displayed the apoptotic activity of 26.4 and 13.9, respectively. The in vitro drug release profiles revealed sustained and controlled PPT release mechanisms, suggesting the stability of the ester bond between the polymer backbone and PPT at pH 5 and 7.4. Furthermore, in vivo antitumor studies of PPT-incorporated polymeric conjugate was performed in mice bearing MCF-7 with tumor volume of 60 mm^3^ and inhibition of tumor growth by increasing the tumor volume to only 146 mm^3^ at the final phase of the treatment, displaying a tumor suppression rate of 82.5% when compared to the free PPT and reference group (PBS) that exhibited 526.7 mm^3^ and 830 mm^3^, respectively [117]. The overexpression of P-glycoprotein (P-gp) is one factor that contributes to MDR. Podophyllotoxin (PPT) inhibits the overexpression of P-gp and the growth of cancer cells. However, it is not used in clinical cancer treatment because of its poor aqueous solubility and toxicity. Incorporating it into polymers resulted in a good therapeutic outcome in vivo, suggesting that polymer-drug conjugates are a promising synthetic approach for chemotherapeutics agents with poor water solubility and toxic nature.

Ndamase et al. prepared polyamidoamine (PAMAM) incorporated with platinum drugs and pamidronate via Michael addition polymerization reaction [118]. The SEM images displayed interwoven and porous morphology images, indicating a controlled and sustained drug release mechanism of the conjugates. The in vitro cytotoxicity studies of the conjugates against HeLa cell lines indicated that the free chemotherapeutics, platinum drugs, and pamidronate were toxic. The PAMAM-platinum conjugate displayed a survival rate at a minimum of 40.67% to a maximum of 67.45%, while the conjugate incorporated with both drugs displayed improved survival rate at a minimum of 55.47 to a maximum of 81.13% [118]. These findings revealed the potential of combining anticancer drugs with other therapeutic agents that are not anticancer agents using a polymer-based drug delivery system.

Hyun and co-workers synthesized beta-cyclodextrin-based-conjugates composed of polyethylene glycol (PEG) and folic acid for enhanced targeted delivery of DOX to breast cancer cells [119]. The DLS and TEM images revealed the conjugates nanosize ranged between 38 nm to 52 nm. The in vitro drug release of the conjugates revealed sustained and controlled release of DOX for 48 h at three different pH solutions of 5.5, 6.8, and 7.4. The controlled release of DOX in the endosome site (that has a pH of 5.5) revealed the potential of polymer-drug conjugates for breast cancer therapy. The in vivo studies were performed on MCF-7 breast cancer-bearing mice, and they displayed decreased tumor volumes of 7.9 to 9.4-fold when compared to the free DOX [119]. The formulation did not cause any severe side effects in vivo, such as cardiotoxicity, etc.

He et al. designed pH-sensitive polymer-drug conjugates by strain-promoted alkyne-azide cycloaddition click reaction incorporated with DOX to afford mPEG-b-norbornene functional PLA-graft-Doxorubicin (mPEG-b-PLA-g-DOX). The Schiff base between the DOX and cyclooctyne enhanced the pH stimuli-responsiveness of the drug for controlled release. The in vitro drug release revealed the slow release of doxorubicin at pH 7.4 with a 19.04% drug release for 140 h. At pH 5.3, the DOX release was 74.73%. The drug release results confirmed the pH-sensitivity of the conjugate and its ability to release the drug at pH 5.3. Furthermore, the cytotoxicity studies showed cytocompatibility of polymeric carriers to MCF-7 breast cancer cell lines when the viability of cells was greater than 80% after the cells exposed to 1000 μg/mL of polymeric carriers [120].

Xu et al. formulated creatine-based polymeric prodrugs, which self-assembled to micellar nanoparticles for the co-delivery of doxorubicin, and bioengineered microRNA against breast cancer cells [121]. The conjugates displayed a size of approximately 180 nm diameter, confirmed by TEM and DLS. The drug release mechanism in a buffer solution of pH 7.4 displayed a slow release of doxorubicin from co-incorporated carriers when compared to carriers loaded with doxorubicin alone. The cytotoxicity evaluations on 4T1.2 breast cancer cell lines displayed significantly improved cytotoxic effect of co-delivery polymer prodrugs, demonstrating the synergistic effect between doxorubicin and bioengineered microRNA. Similar results were observed in MDA-MB-231 breast cancer cells. Furthermore, in vivo studies displayed that these co-delivery carriers inhibited tumor growth of 4T1.2 metastasis [121].

Chen and co-workers synthesized stimuli-responsive polymer-drug conjugate incorporated with doxorubicin using *N*-(1,3-dihydroxypropan-2-yl) methacrylamide (DHPMA) as the polymers [122]. This polymer-drug conjugate self-assembled to produce nanoparticles with a size of 21 nm and displayed good stability. The conjugate displayed an improved antitumor activity against 4T1 breast tumor with reduced side effects, revealing its safe use as an anticancer agent. The drug release profiles showed slow release of doxorubicin from the conjugate at pH 7.4 when compared to the pH of 5.4 with a 19.7% and 15.2% of doxorubicin released from polymer backbone in 1 h incubation with or without cathepsin B, respectively. The release profile of DOX also confirmed the stability of the hydrazone linker between doxorubicin and the polymer backbone. Furthermore, the cytotoxicity studies of conjugate and free doxorubicin against breast cancer cells demonstrated an IC_50_ value of 0.79 µg/mL and 0.31 µg/mL, respectively [122]. The conjugates’ blood circulation time was extended with an elimination half time of 9.8 h, indicating high uptake in the tumors. The in vivo therapeutic efficacy against 4T1 xenograft tumors was significant when compared to the free DOX. The tumor inhibition was via the inhibition of antiangiogenic effects and cell proliferation. The uptake of the conjugate-based nanoprodrug was via endocytosis, resulting in the release of DOX, which disrupted the mitochondrial functions and cellular apoptosis. Furthermore, conjugates with a high molecular weight of 95 kDa displayed an extended blood circulation time with significant accumulation in the tumors [122].

Ding et al. synthesized polyethylene glycol (PEG) and poly (β-l-malic acid)-drug conjugates by covalently incorporating anti-HER2/neu peptide (AHNP) (trastuzumab-mimetic 12-merpeptide) for the treatment of HER2-positive breast cancer. The in vivo tumor growth inhibition studies against HER2-positive breast cancer was performed using phosphate buffer saline (PBS) as a control, free AHNP, non-loaded nanoconjugate, and AHNP-incorporated conjugates via intravenous administration in nude mice inoculated with BT474 breast cancer cells (HER2-positive), which developed to the tumor with a size of 120 mm^3^ [123]. The in vivo assessments revealed that after injection with non-loaded nanoconjugate, the tumor growth was similar to the tumor growth in nude mice that were injected with PBS after eight injections a month (two injections a week). Significant tumor growth inhibition was observed after injection with AHNP-loaded conjugate, which decreased the tumor size about 20 times when compared to PBS [123].

Aderibigbe et al. prepared a class of polymer-drug conjugates containing bisphosphonates, platinum, and DNA-demethylating agent [124]. The conjugates, containing alendronate alone, did not display any cytotoxic effect against the cancer cell lines, MDA-MB-231 and MCF-7. The drug release studies of the conjugates containing the only alendronate revealed a slow drug release of approximately 10.9% and 11.9% of alendronate from one of the conjugates at pH 1.2 and 7.4, respectively. For 7 days, only 17.6% and 18.9% of alendronate were released at pH 1.2 and 7.4, respectively, from the conjugate. The slow-release profile of bisphosphonate from the backbone of the carrier might have contributed to the non-cytotoxic effects of the conjugate containing the only alendronate on the cancer cell lines. The conjugate containing platinum (II), procaine, and alendronate displayed a potent cytotoxic effect against the cancer cell lines. The free drugs displayed a high toxic effect when evaluated against a normal cell line, EA. hy926 cells when compared to the conjugates. The conjugates were water-soluble and degradable at physiological pH [124]. In another research report by Aderibigbe et al., polyaspartamide-based conjugates, containing ferrocene and platinum (II) drug, were prepared. The average particle charges were 29 and 30.2, revealing their good stability and capability to resist aggregation. In vitro cytotoxicity studies further revealed that the conjugates did not exhibit cytotoxic effect towards the normal cell lines (EA. hy926), indicating their non-toxic effect. However, they exhibited a high cytotoxic effect against the cancer cell lines—MCF-7 and MDA-MB-231—which further confirmed their good selectivity towards cancer cell lines when compared to the normal cell lines. The in vitro drug release studies were sustained [125].

Several research reports of polymer-drug conjugates designed for the treatment of breast cancer displayed unique features, such as high cytotoxic effects against the breast cancer cell lines [111,112], suitable for the combination of two anticancer drugs, resulting in synergistic effects [112,113,115,121,124,125], high selectivity towards cancer microenvironment, followed by tumor cell death and increased the survival rate in vivo [115], extended blood circulation [122], extended half-life [116], good stability [124,125], and improved water solubility of the incorporated drug [117]. However, in one of the reports, the design of polymer-drug conjugates containing a single anticancer drug did not produce a significant cytotoxic effect in vivo when compared to polymer-drug conjugates containing two anticancer drugs [115].

Some factors have contributed to the high efficacy of polymer-drug conjugates for the treatment of breast cancer. The use of enzyme-sensitive linkers has resulted in the cleavage of the linkers, followed by the sustained release of the drug in the cancer microenvironment [111]. The incorporation of drugs via short length hydrazone spacers has enhanced the anticancer activity of the conjugates [115]. The molecular weight and branching of the conjugates have influenced the drug retention in the mammary gland and cytotoxic effect [116]. Incorporation of anticancer drugs together with drugs, which are not anticancer agents, has resulted in synergistic effects, such as bisphosphonates [118,124], DNA-demethylating agent [124], and folic acid [119]. Conjugates with a high molecular weight of 95 kDa have displayed an extended blood circulation time with significant accumulation into the tumors [122]. The pH stimuli-responsiveness of the conjugates has contributed to their controlled release mechanism [120]. The nature of the bond between the polymer backbone and the drug, such as ester bond, has resulted in sustained drug release at simulated physiological pH [117]. The nature of the polymer used plays a significant role in the efficacy of the conjugates and route of administration [112]. Hyaluronic acid capability to degrade easily in the lymphatic system has revealed that the subcutaneous administration of the hyaluronic acid-based conjugates has resulted in the reduction of the molecular weight before its uptake into the systemic circulation. The subcutaneous administration of the conjugates in the mammary fat pads has induced the draining of the conjugates into the lymph nodes, a site of early tumor metastases [112]. The route of administration of the conjugates, such as intraductal administration, has prolonged the ductal retention of the conjugates [116].

## 5. Polymer-Anticancer Drug Conjugates for Lung Cancer Treatment

Lung cancer is an infectious lung tumor, which is also known as lung carcinoma. It is caused by uncontrollable cell growth in the tissues of the lungs. The cell growth spreads by a process known as metastasis to other parts of the body. There are two types of primary lung cancers: small-cell lung cancer (SCLC) and non-small-cell lung cancer (NSCLC) [126,127]. Common symptoms of lung cancer include weight loss, chest pains, short breath, coughing (sometimes coughing blood), etc. [128]. An estimated 85% majority of patients diagnosed with lung cancer are due to long-term smoking, whereas about 10–15% of patients diagnosed with lung cancer never smoked [129]. These cases are often caused by a combination of genetic factors and exposure to radon gas, second-hand smoke, asbestos, or exposure to air pollution, etc. Lung cancer can be diagnosed by chest radiographs and computed tomography (CT) scans. Biopsy also confirms the diagnosis of lung cancer, and it is usually performed by bronchoscopy or CT-guidance [130]. The anticancer drugs used to treat lung cancer suffer from limitations, which can be overcome by polymer-drug conjugates. Some researchers reported the efficacy of polymer-drug conjugates for lung cancer treatment in vitro and in vivo (Table 3).

Kumar et al. synthesized hyaluronic acid conjugate incorporated with dihydroartemisinin, which is a well-known antimalarial agent via covalent ester linker for the treatment of lung cancer [131]. The physiochemical properties and the successful incorporation of dihydroartemisinin to hyaluronic acid was confirmed by FTIR, ^1^H NMR, and GPC. The percentage incorporation of dihydroartemisinin in the conjugate was 12.33%. The conjugate self-assembled in aqueous media to form nanoparticles with a particle size of 267.6 ± 11.29 nm and PDI 0.22 ± 0.01 revealed by DLS. The in vitro cytotoxicity efficacy of the conjugate and dihydroartemisinin was evaluated against lung cancer cells (A549) utilizing CCK8 assay. The cytotoxicity outcome was superior for the conjugate when compared to the free dihydroartemisinin at a concentration of 1 μg/mL. Furthermore, the apoptosis studies demonstrated that lung cancer cells treated with conjugate induced higher apoptosis when compared to the free dihydroartemisinin, confirming the potential of polymer-drug conjugates [131].

Chen prepared paclitaxel-polymeric prodrug by incorporating paclitaxel to hyaluronic acid via C-6 of *N*-acetyl-d-glucosamine utilizing hexanediamine linker [132]. The polymeric prodrug with a paclitaxel loading capacity of 21.8% exhibited a good aqueous solubility of 168 mg/mL and displayed an increased drug release mechanism in the presence of an enzyme (hyaluronidase). The drug release mechanism of paclitaxel from the conjugate was slow at pH 7.5, with less than 20% of paclitaxel for 96 h. The drug release profile was slower at pH 6, with less than 12% of paclitaxel released after 96 h. Hence, the drug release mechanism from hyaluronic acid can be increased by naturally occurred hyaluronidase that can be favored at acidic conditions that range between pH 5.5 and 6.

The in vitro cytotoxicity studies of the conjugate displayed superior antitumor activity, after 2 days, against A549 lung cell lines when compared to the free paclitaxel. Furthermore, the apoptosis studies on the conjugate treatment group and free paclitaxel treatment group exhibited good apoptosis percentage of A549 lung cells of 30.46% and 7.11% after 2 days of treatment, respectively, when compared to 5.50% of the control group. The conjugate treatment group displayed a significant increase in the proportion of necrotic and apoptotic cells when compared to the free paclitaxel treatment group [132].

Wang et al. prepared polymer-drug conjugates loaded with paclitaxel and doxorubicin, resulting in synergistic anticancer activity, using a novel biodegradable and amphiphilic biodegradable triblock copolymer called mPEG-b-norbornene functional PLA-b-P(α-BrCL) [133]. An adjustment of the length of polycaprolactone and polylactic acid demonstrated that this polymer could have a relatively sufficient quantity of 15.8 wt% of doxorubicin and 12.1 wt% of paclitaxel, respectively. The physiochemical properties and successful preparations of the polymer-dual drug conjugates were confirmed by HPLC and ^1^H NMR. The in vitro drug release profiles displayed fast doxorubicin release at pH 5, which was about 52.20% caused by acidic pH sensitivity of the amide linker when compared to pH 7.4, which was about 13.76% after 80 h; while the drug release profiles of paclitaxel release from the polymers displayed slow, sustained, and controlled release mechanism at pH 7.4 and 5 with the quantity of 29.85% and 38.25%, respectively.

The in vitro cytotoxic studies revealed cell viability above 85% when a blank carrier was incubated with A549 lung cell lines at a concentration up to 100 mg/mL after 72 h, indicating the low toxicity of the carriers against A549. The free anticancer drugs and polymeric prodrugs showed clear mortality in A549 lung cell lines. The IC_50_ values of paclitaxel-incorporated polymers and doxorubicin-incorporated polymers were 0.25 μg/mL and 2.60 μg/mL, respectively. In addition, the IC_50_ value of dual polymer-drug conjugates was lower than 1, indicating a clear synergistic effect [133]. The incorporation of both drugs into the carrier resulted in a synergistic effect in inhibiting the proliferation of A549 cancer cells.

Sun et al. synthesized sulfobetaine polymer-paclitaxel conjugates using allyl-functionalized polylactide as the polymer backbone precursor to afford zwitterionic biodegradable polymer prodrugs [134]. These polymer prodrugs without paclitaxel did not display any considerable cytotoxic activity up to a concentration of 1000 µg/mL. The polymer prodrug incorporated with paclitaxel demonstrated high anticancer activity against A549 lung cancer cells when compared to the free paclitaxel at paclitaxel concentration that is higher than 1 µg/mL. The incubation of cancer cells with conjugates containing 10 µg/mL showed a low cell viability of 20% in A549 cells after 72 h, confirming anticancer efficacy. The release of paclitaxel from the polymer backbone was not complete in 3 days at pH 7.4. Furthermore, paclitaxel release at pH 5.5 was 11.4% in the first 4 h and 1.6% at pH 7.4. After 120 h, the release of paclitaxel from the polymer backbone was less noticeable. However, 83.5 ± 4.9% and 72.7 ± 4.1% of the drug was released at pH 5.5 and 7.4, respectively.

Luo et al. synthesized polymer-drug conjugates incorporated with paclitaxel via two selected linkers for lung cancer targeting using polyethylene glycol (PEG) as the polymers. These PEG conjugates significantly increased the water solubility of paclitaxel up to 4-fold when compared to the free paclitaxel [135]. The conjugates were stable at pH 6.9 (pH of the human lung lining fluid) when compared to pH 7.4 (pH of human blood). The in vitro cytotoxicity studies of the polymer-drug conjugates was assessed on both LL/2 Lewis lung carcinoma cells and B16-F10 melanoma cells, which are usually utilized models in mice to evaluate the anticancer efficacy of chemotherapeutics. The PEG conjugates displayed cytotoxic effect on both lung cancer models but lower than the free paclitaxel (used as control) and taxol^®^ (commercially and clinically used paclitaxel). The IC_50_ values of the conjugates was 0.0286 μg/mL against LL/2 cells and 0.0521 μg/mL against B16-F10 cells when compared to taxol^®^ (0.0039 μg/mL against LL/2 cells and 0.0100 μg/mL against B16-F10 cells) and free paclitaxel (0.0022 μg/mL against LL/2 cells and 0.0069 μg/mL against B16-F10 cells), respectively [135]. The cytotoxic effect of the conjugates was low when compared to the free drugs.

Shamay et al. prepared vascular endothelial growth factor receptor (VEGFR)-1-targeted *N*-(2-hydroxypropyl)methacrylamide (HPMA) copolymer-drug conjugate incorporated with doxorubicin. The binding and internalization profiles of HPMA-doxorubicin conjugate indicated that this conjugate is superior to endothelial cells when compared to cancer cells [136]. The cytotoxicity studies of the polymer-drug conjugate indicated an IC_50_ value of 0.15 μM against bEnd.3 cells, which was 370-fold cytotoxic when compared to the peptide-doxorubicin. Furthermore, the conjugate displayed high cytotoxic activity against the lung cancer cells, which was 10-fold higher cytotoxic against B16-F10, 3LL, and HT29 cells when compared to peptide-doxorubicin [136].

Yang et al. formulated pH/reduction-responsive polymer-drug conjugates using poly-l-lysine-lipoic acid (PLL-LA) incorporated with doxorubicin. The in vitro drug release studies of polymeric prodrugs exhibited sustained doxorubicin release at a physiological pH of 7.4, and a fast doxorubicin release mechanism was influenced by the tumor extracellular pH of 6.8, leading to enhanced internalization. These carriers displayed a superior ability of tumor-targeting in A549 tumor tissues as well as antitumor activity. Furthermore, the cytotoxicity studies of the carriers incorporated with doxorubicin displayed superior cytotoxic effect against A549 cells at pH 6.8 when compared to pH 7.4 [137].

Polymer-drug conjugates designed for the treatment of lung cancer have been reported to significantly enhance the water solubility of the conjugated drug [132]. A synergistic effect has also been reported for polymer-drug conjugates containing two anticancer drugs when compared to the conjugates with a single drug [133]. The conjugates are reported to be highly cytotoxic when compared to free drugs [135]. Polymer-drug conjugates have been reported to exhibit significant tumor-targeting capability when compared to the free drug [137]. The aforementioned features make them potential therapeutics, which can overcome drug toxicity, which is common with conventional anticancer drugs.

## 6. Conclusions

Polymer-drug conjugates are promising polymer-based carriers that can be used for the treatment of cancer. Some polymer-drug conjugates are currently in clinical trials. The first polymer-drug conjugate that entered clinical trial was *N*-(2-hydroxypropyl) methacrylamide copolymer (HPMA)-doxorubicin (PK1), about 20 years ago. The recently reported polymers for the preparation of polymer-drug conjugates for the targeting of breast and lung cancer cells are hyaluronic acid, *N*-(2-hydroxypropyl)methacrylamide (HPMA), poly (ethylene) glycol (PEG) because of their distinct physicochemical properties. These properties include their good biocompatibility, low immunogenicity, and prolonged circulation of an incorporated drug. The dual combination therapy using polymer-drug conjugates promotes a synergistic anticancer effect. Factors, such as the nature of the linker, the design of the conjugates, the molecular weight, the size, play a huge role in the biological efficacy of the prepared conjugates. In the last four years, most of the conjugates developed contained two anticancer drugs resulting in synergistic effects. The in vivo studies were promising and revealed the potential of polymer-drug conjugates. However, there are also some limitations of polymer-drug conjugates, which have not been thoroughly studied, such as the behavior of polymer-drug conjugates in blood. The more study is performed on how these systems interact with biology in the nanoscale, the closer some of these systems would reach the clinical stage. There is no doubt that continuous research in the development of nanocarriers would result in potent therapeutics for the treatment of cancer.

## Figures and Tables

**Table 1:** A summary of the classification of anticancer drugs.

Classes of Anticancer Drugs	Mode of Action	General Mechanisms of Resistance	Examples
Topoisomerase inhibitors	They hinder the binding of the DNA substrate. They also form a cleavage complex, which prevents enzyme turnover and the build-up of high levels of the cytotoxic cleavage complex within the cell.	The altered proliferation and drug targets, reduced sensitivity to apoptosis and cell death, increased ability to repair DNA damage, expression of drug efflux pumps, and detoxification mechanisms.	**1–3**
Antimetabolites	They hinder the biosynthesis of nucleic acids.		**4–7**
Anti-tubulin agents	They disrupt mitotic spindles and terminate mitosis.		**8–11**
Alkylating agents	They bind covalently with the DNA and crosslink them, thereby disrupting the DNA.		**12–16**

**Table 2:** Summary of polymer-drug conjugates efficacy on breast cancer in vitro and in vivo.

Polymer-Drug Conjugates	Carrier/ Monomers Used	Drugs	Biological Outcomes	Molecular Design	Reference
*N*-(2-hydroxypropyl methyl) acrylamide copolymer-gadolinium-paclitaxel-Cyanine5.5	*N*-(2-hydroxypropyl methyl) acrylamide	Paclitaxel	Mode of administration in vivo: Intravenous. Prolonged residence time, high accumulation of the conjugate at the tumor site. Inhibition of proliferation and induced apoptosis of the 4T1 murine breast cancer cells.	The amphiphilic block polymer was prepared via a two-step Reversible Addition-Fragmentation chain Transfer polymerization and self-assembled into a nanoparticle. Enzyme-sensitive tetrapeptide linker was used as a spacer in the conjugate to promote the degradation of high molecular weight conjugates into low molecular weights with the release of the drug in the cancer microenvironment.	[111]
Hyaluronic acid-Doxorubicin-Gemcitabine	Hyaluronic acid	Doxorubicin and gemcitabine	Mode of administration in vivo: Intravenous and subcutaneous. The conjugates loaded with both drugs were active in inhibiting the formation of an orthotopic, aggressive 4T1 tumor model in vivo when compared to individual drugs and the polymer-conjugates loaded with a single drug.	The amine on gemcitabine was conjugated to the carboxylic acid on amino acids to form a prodrug. The prodrug was conjugated to hyaluronic acid via carbodiimide chemistry. Doxorubicin was conjugated to hyaluronic acid via carbodiimide chemistry.	[112]
PEG-folic acid-trastuzumab	Polyethylene glycol	Folic acid and trastuzumab	The in vitro cellular uptake of the prodrugs conjugated with both drugs was high when compared to the non-targeted polymeric prodrugs. The conjugate displayed apoptosis of 80% with enhanced tumor regression in vivo.	The copolymer was prepared by ring-opening polymerization of PEG and lactide followed by isomerization polymerization of the triblock copolymer and 2-hydroxyethyl disulfide using dibutyltin dilaurate as a catalyst.	[113]
*N*-(2-hydroxypropyl) methacrylamide -Doxorubicin	*N*-(2-hydroxypropyl) methacrylamide	Doxorubicin	Mode of administration in vivo: Intravenous. The conjugate exhibited reduced glycolysis, increased apoptosis, and reduced degree of phospholipids when compared to the free doxorubicin. The in vivo studies on the 4T1 breast tumor mouse model using the conjugate revealed a high reduction in the growth of tumors when compared to free DOX-treated mice.	DOX was incorporated into the carriers, and enzyme-sensitive tetrapeptide linker was used as a spacer in the conjugate to promote the degradation of high molecular weight conjugates into low molecular weights with the release of the drug in the cancer microenvironment.	[114]
Poly-l-glutamic acid-Doxorubicin-Aminoglutethimide	Poly-l-glutamic acid	Doxorubicin and aminoglutethimide	Mode of administration in vivo: Intravenous. The conjugates displayed enhanced tumor cell death and inhibited tumor-related activities. However, the conjugates containing [*N*-ε-maleimidocaproic acid hydrazide] moiety displayed a higher survival rate and pro-apoptotic activity, lower anti-apoptotic signals, and inhibition of metastasis.	The conjugates loaded with Dox and aminoglutethimide were prepared with pH-sensitive linkers—hydrazine moiety or complex EMCH [*N*-ε-maleimidocaproic acid hydrazide] moiety—for the release of Dox in the tumor microenvironment.	[115]
Polyethylene glycol -Doxorubicin (PEG-DOX)	Polyethylene glycol	Doxorubicin	Mode of administration in vivo: Intraductal. Increased molecular weight and decreased branching prolonged the retention of the drug in the mammary gland after administration.	Dox was conjugated to PEG polymers with varied molecular weights (5, 10, 20, and 40 kDa) and architectures of linear, four-arm, and eight-arm.	[116]
Poly(l-glutamic acid)-g-methoxy poly(ethylene glycol) (PLG-g-Mpeg-PTT	Poly(l-glutamic acid)-g-methoxy poly(ethylene glycol)	Podophyllotoxin	The conjugates decreased the hemolytic activity of the drug. The conjugates’ antitumor activity against MCF-7/ADR xenograft tumors was high, with a tumor suppression rate of 82.5%.	The drug was conjugated into poly(l-glutamic acid)-g-methoxy poly(ethylene glycol) (PLG-g-mPEG) via ester bonds.	[117]
Polyamidoamine-Pamidronate-Platinum (PAMAM-PAM-Pt)	Polyamidoamine	Pamidronate and platinum	The conjugates were not toxic when compared to the free drugs.	The conjugates were synthesized by aqueous phase Michael-addition polymerization reaction.	[118]
Beta-cyclodextrin- Polyethylene glycol-Folic Acid-doxorubicin (β-CD-PEG-FA-DOX)	Polyethylene glycol, Beta-cyclodextrin	Doxorubicin	Mode of administration in vivo: Intravenous. Reduced tumor volume, no systemic toxicity, and cardiotoxicity.	Beta-cyclodextrin (β-CD)-based carrier was composed of β-CD, polyethylene glycol, and folic acid for enhanced drug delivery.	[119]
Methoxy Polyethylene glycol-Polylactic acid-Doxorubicin (mPEG-b-PLA-g-DOX)	Polyethylene glycol, Polylactic acid	Doxorubicin	The cytotoxicity studies showed the cytocompatibility of polymeric carriers to MCF-7 breast cancer cell lines with the viability of cells greater than 80%.	The conjugates were prepared by ring-opening polymerization and condensation followed by click reaction. The carriers were grafted with a triazo group. Doxorubicin was modified with cyclooctyne and conjugated to the carriers by strain-promoted alkyne-azide cycloaddition click reaction.	[120]
poly(oligoethylene glycol acrylate) (POEG-VBC-DOX)	poly(oligoethylene glycol acrylate)	Doxorubicin	Mode of administration in vivo: Intravenous. Synergistic anti-tumor and anti-metastasis activity in vitro and in vivo.	DOX was incorporated POEG-VBC backbone.	[121]
*N*-(1,3-dihydroxypropan-2-yl) methacrylamide-Doxorubicin	*N*-(1,3-dihydroxypropan-2-yl) methacrylamide	Doxorubicin	Extended blood circulation time with an elimination half time of 9.8 h. High accumulation in the tumors and improved in vivo therapeutic efficacy against 4T1 xenograft tumors compared to the free DOX. Tumor inhibition was via inhibition of cell proliferation and antiangiogenic effects.	The conjugates were synthesized by RAFT polymerization, followed by drug conjugation.	[122]
Polymalic acid-Trastuzumab	Polymalic acid	Trastuzumab	Mode of administration in vivo: Intravenous. Enhanced tumor growth inhibition.	Polyethylene glycol (PEG) and poly (β-l-malic acid)-drug conjugates were prepared by covalently incorporating anti-HER2/neu peptide.	[123]
Polyamidoamine-Procaine-Platinum-Alendronate	Polyamidoamine	Procaine, Platinum (II), Alendronate	Selective inhibitory effects of the conjugates towards the cancer cell lines.	The conjugates were synthesized by aqueous phase Michael-addition polymerization reaction.	[124]
Polyamidoamine-Procaine-Pt-Alendronate	Polyamidoamine	Ferrocene, Pt (II)	Selective inhibitory effects of the conjugates towards the cancer cell lines.	The conjugates were synthesized by aqueous phase Michael-addition polymerization reaction.	[125]

**Table 3:** Summary of polymer-drug conjugates, which are effective in vitro and in vivo against lung cancer.

Polymer-Drug Conjugates	Carrier/Monomers Used	Drugs	Biological Outcomes	Molecular Design	Reference
Hyaluronic acid-dihydroartemisinin (HA-DHA)	Hyaluronic acid	Dihydroartemisinin	The conjugates displayed high apoptosis when compared to the free drug	The hydroxyl group of the drug was covalently linked to the carboxylic group of hyaluronic acid.	[131]
Hyaluronic acid-Paclitaxel (HA-PLX)	Hyaluronic acid	Paclitaxel	Significant cytotoxicity and apoptosis-inducing effect resulting from increased cellular uptake of the drug via HA-receptor mediated endocytosis.	Paclitaxel was conjugated to the C-6 position of N-acetyl-D-glucosamine of the hyaluronic acid using hexanediamine as a linker.	[132]
MPEG-b-norbornene functional PLA-b-P(α-BrCL)	Polylactic acid, Polyethylene glycol	Doxorubicin and paclitaxel	The incorporation of both drugs into the carrier resulted in a synergistic effect in inhibiting the proliferation of A549 cancer cells.	Both drugs were covalently incorporated into the polymer backbone	[133]
Polylactide-Paclitaxel (PLA-PTX)	Allyl-functionalized polylactide	Paclitaxel	Enhanced cytotoxic effect in vitro.	A polymer-drug conjugate was also obtained by thiol-ene reaction of both thiol-functionalized SB and PTX with allyl-functionalized PLA.	[134]
Polyethylene glycol-Paclitaxel (PEG-PTX)	Polyethylene glycol	Paclitaxel	The conjugates exhibited sustained drug release with anti-tumor activity, which was less than the free drugs.	The conjugates were prepared with either an azide linker or a succinic linker. The linear PEGs were modified with PTX at the hydroxyl. PTX was incorporated into the PEG molecule via an ester bond at the C-2′ position on the PTX side chain.	[135]
*N*-(2-hydroxypropyl)methacrylamide-Doxorubicin	*N*-(2-hydroxypropyl)methacrylamide	Doxorubicin	High cytotoxic activity against the lung cancer cells, which were 10-fold higher cytotoxic against B16-F10, 3LL, and HT29 cells when compared to peptide-doxorubicin.	Doxorubicin was incorporated into *N*-(2-hydroxypropyl)methacrylamide.	[136]
Poly-l-lysine-lipoic acid-Doxorubicin	Poly-l-lysine-lipoic acid	Doxorubicin	The conjugates exhibited enhanced internalization and cytotoxicity effects in vitro. It also exhibited excellent good tumor-targeting capability.	It was prepared by the modification of dimethylmaleic anhydride for enhanced cell internalization	[137]
